# The *APOBEC3B* c.783delG Truncating Mutation Is Not Associated with an Increased Risk of Breast Cancer in the Polish Population

**DOI:** 10.3390/genes14071329

**Published:** 2023-06-24

**Authors:** Katarzyna Gliniewicz, Wojciech Kluźniak, Dominika Wokołorczyk, Tomasz Huzarski, Klaudia Stempa, Helena Rudnicka, Anna Jakubowska, Marek Szwiec, Joanna Jarkiewicz-Tretyn, Mariusz Naczk, Tomasz Kluz, Tadeusz Dębniak, Jacek Gronwald, Jan Lubiński, Steven A. Narod, Mohammad R. Akbari, Cezary Cybulski

**Affiliations:** 1 International Hereditary Cancer Center, Department of Genetics and Pathology, Pomeranian Medical University in Szczecin, 71-252 Szczecin, Poland; katarzyna.gliniewicz@pum.edu.pl (K.G.); wojciech.kluzniak@pum.edu.pl (W.K.); dominikawok@o2.pl (D.W.); tomasz.huzarski@pum.edu.pl (T.H.); klaudia.stempa@pum.edu.pl (K.S.); helena.rudnicka@pum.edu.pl (H.R.); aniaj@pum.edu.pl (A.J.); tadeusz.debniak@pum.edu.pl (T.D.); jacek.gronwald@pum.edu.pl (J.G.); jan.lubinski@pum.edu.pl (J.L.); 2 Department of Clinical Genetics and Pathology, University of Zielona Góra, 65-046 Zielona Góra, Poland; 3 Independent Laboratory of Molecular Biology and Genetic Diagnostics, Pomeranian Medical University in Szczecin, 70-204 Szczecin, Poland; 4 Department of Surgery and Oncology, University of Zielona Góra, 65-046 Zielona Góra, Poland; m.szwiec@cm.uz.zgora.pl; 5 Non-Public Health Care Centre, Cancer Genetics Laboratory, 87-100 Toruń, Poland; jarkiewicztretyn@poczta.onet.pl; 6 Institute of Health Sciences, Collegium Medicum, University of Zielona Góra, 65-417 Zielona Góra, Poland; m.naczk@inz.uz.zgora.pl; 7 Department of Gynecology and Obstetrics, Institute of Medical, Sciences, Medical College of Rzeszów University, 35-959 Rzeszów, Poland; jtkluz@interia.pl; 8 Women’s College Research Institute, Women’s College Hospital, Toronto, ON M5S 1B2, Canada; steven.narod@wchospital.ca (S.A.N.); mohammad.akbari@utoronto.ca (M.R.A.); 9 Dalla Lana School of Public Health, University of Toronto, Toronto, ON M5T 3M7, Canada

**Keywords:** Polish population, *APOBEC3B* gene, mutation, breast cancer risk

## Abstract

The *APOBEC3B* gene belongs to a cluster of DNA-editing enzymes on chromosome 22 and encodes an activation-induced cytidine deaminase. A large deletion of *APOBEC3B* was associated with increased breast cancer risk, but the evidence is inconclusive. To investigate whether or not *APOBEC3B* is a breast cancer susceptibility gene, we sequenced this gene in 617 Polish patients with hereditary breast cancer. We detected a single recurrent truncating mutation (c.783delG, p.Val262Phefs) in four of the 617 (0.65%) hereditary cases by sequencing. We then genotyped an additional 12,484 women with unselected breast cancer and 3740 cancer-free women for the c.783delG mutation. The *APOBEC3B* c.783delG allele was detected in 60 (0.48%) unselected cases and 19 (0.51%) controls (OR = 0.95, 95% CI 0.56–1.59, *p* = 0.94). The allele was present in 8 of 1968 (0.41%) familial breast cancer patients from unselected cases (OR = 0.80, 95% CI 0.35–1.83, *p* = 0.74). Clinical characteristics of breast tumors in carriers of the *APOBEC3B* mutation and non-carriers were similar. No cancer type was more frequent in the relatives of mutation carriers than in those of non-carriers. We conclude the *APOBEC3B* deleterious mutation p.Val262Phefs does not confer breast cancer risk. These data do not support the hypothesis that *APOBEC3B* is a breast cancer susceptibility gene.

## 1. Introduction

Breast cancer is the most common malignancy among women. Annually, it is diagnosed in over two million women worldwide, including about 20,000 cases in Poland. The lifetime risk of breast cancer in the Polish population is approximately 8% [1]. However, this risk varies by lifestyle, environmental and genetic factors, including the presence of predisposing mutations in breast cancer susceptibility genes. The risk of breast cancer is higher in families with strong clustering of breast cancer than in those with no or single affected relatives [2].

Approximately 10–15% of all cases of breast cancer are hereditary, and these cases are caused by mutations in high or moderately penetrant breast cancer susceptibility genes. To date, over twenty genes have been associated with a genetic predisposition to breast cancer. Based on the mutation frequencies, the magnitude of cancer risk, and the influence on clinical management with mutation carriers, the most important breast cancer susceptibility genes include *BRCA1*, *BRCA2*, *PALB2*, and *CHEK2* [3,4,5,6,7,8]. Other genes which have been associated with breast cancer susceptibility of moderate penetrance, and their mutations are relatively common among breast cancer cases, are *ATM*, *BARD1*, *RAD51C*, and *RAD51D*, but the importance of identifying pathogenic mutations of these genes in clinical practice has not been well established [9,10,11,12,13,14]. Other breast cancer susceptibility genes such as *TP53*, *PTEN*, *STK11*, and *CDH1* are reported to confer a high or moderate increase in the risk of breast cancer, but mutations of these genes are very rare [15,16,17,18]. For several other genes, including *BLM*, *NBN*, *XRCC2*, *RECQL, BRIP1*, and *RAD50*, the evidence for an association with breast cancer is weak or mixed [19,20,21,22,23,24]. 

In 2021, the results of two case–control studies that tested the associations between putative cancer susceptibility genes and the risk of breast cancer were reported. The first report analyzed 34 genes and 113,000 women from 25 countries, and the second study investigated 28 genes in 64,000 women from the United States [24,25]. Variants in *BRCA1*, *BRCA2*, *PALB2*, *BARD1*, *RAD51C*, *RAD51D*, *ATM*, *CHEK2*, and *TP53* showed significant associations with an increased risk of breast cancer in both studies. However, together, pathogenic mutations in all known breast cancer predisposing genes are responsible for about 10% of unselected cases of breast cancer, and are detected in approximately half of the families with strong aggregation of breast cancer (families with hereditary breast cancer, HBC families). 

Other breast cancer susceptibility genes are likely to exist. Most recently, in 2023, we identified a novel gene for hereditary breast cancer called *ATRIP*. We detected *ATRIP* c.1152_1155del (p.Gly385Ter) founder mutation in 42 of 16,085 unselected Polish breast cancer patients and in 11 of 9285 cancer-free women (OR = 2.14, 95% CI 1.13–4.28, *p* = 0.02). By analyzing the sequence data of the UK Biobank, we identified *ATRIP* loss of function variants in 13 of 15,643 breast cancer patients and 40 of 157,943 female controls (OR = 3.28, 95% CI = 1.76–6.14, *p* < 0.001). In addition, we performed immunohistochemistry and functional studies that showed weak expression of the allele with the *ATRIP* c.1152_1155del variant compared to the wild-type allele, and we observed that truncated ATRIP fails to perform its function to prevent replicative stress. We also showed that breast cancers in women who carry a germline *ATRIP* mutation have a loss of heterozygosity at the site of the mutation and a deficiency in DNA repair by homologous recombination [26]. 

*APOBEC3B* is a good candidate gene for breast cancer susceptibility. The APOBEC3B (Apolipoprotein B mRNA editing enzyme catalytic subunit 3B) belongs to a family of proteins called activation-induced cytidine deaminases (AICDA/AID), including APOBEC3A to APOBEC3G, which are encoded by an *APOBEC* genomic cluster on chromosome 22 and are known to protect human cells from viral infections by inducing mutations of single-stranded DNA [27]. In detail, DNA cytidine deaminases act as inhibitors of retrovirus replication and retrotransposon mobility via deaminase-dependent and -independent mechanisms. After the penetration of retrovirus into cells and the initiation of reverse transcription, they can induce the conversion of cytosine to uracil in the minus-sense single-strand viral DNA, which leads to G-to-A hypermutations in the plus-strand viral DNA. The resultant mutations in the proviral genome, along with a deamination-independent mechanism, exert an antiretroviral effect in infected target cells [28]. 

A link between the *APOBEC3* genes and DNA damage was first reported over two decades ago when it was established that the APOBEC3A protein is capable of hypermutating nuclear DNA and inducing double-stranded DNA breaks [29]. Then, it has been shown that also the APOBEC3B protein is able to edit genomic DNA, and has the capability of introducing mutations in DNA [30]. It was reported that APOBEC3B is involved in the immune response against viruses, regulation of DNA methylation, and CHEK2-inducing DNA damage, which leads to apoptosis [31,32]. APOBEC3B has been demonstrated to induce somatic mutations in several types of malignancies, including breast cancer [32]. It has been described as a strong driver of breast cancer [33]. Expression of *APOBEC3B* is upregulated in breast cancer and it was associated with kataegis, aggressive clinical and pathological features of breast tumors, and poor prognosis among patients with estrogen receptor-positive (ER+) breast cancer [34]. Expression of *APOBEC3B* may also be required for estrogen receptor function [35]. A large germline deletion of the *APOBEC3B* gene was reported as a low-risk variant for breast cancer [36,37,38,39,40], but negative reports have also been published [41,42,43,44].

Although overexpression of *APOBEC3B* has been associated with the pathogenesis of breast cancer, the role of germline mutations of this gene in the etiology of breast cancer remains unclear. To investigate whether or not germline intragenic mutations of *APOBEC3B* are present the Polish population and predispose to breast cancer, we studied approximately 13,000 patients with breast cancer and 3700 controls. First, we screened the entire *APOBEC3B* gene in 617 Polish families with hereditary breast cancer by whole exome sequencing and identified a truncating founder mutation c.783delG (p.Val262Phefs). Then, we genotyped approximately 12,500 unselected cases of breast cancer and 3700 cancer-free controls for the c.783delG founder mutation and compared the clinical characteristics of breast tumors in carriers of the *APOBEC3B* mutation with cancers diagnosed among non-carriers. To investigate whether the *APOBEC3B* c.783delG mutation predisposes to different cancers, we reviewed the pedigrees of patients with breast cancer who carry the mutation and compared them with the pedigrees of patients without the mutation.

## 2. Materials and Methods

### 2.1. Hereditary Breast Cancer Cases

For the first step, we selected 617 unrelated breast cancer patients from 617 Polish families with a family history of breast cancer. We selected women with a strong family history of breast cancer (in first- or second-degree relatives). Among the 617 probands with breast cancer, 160 women were from families with at least 4 women affected with breast cancer, 378 women were from families with 3 affected, and 79 women were from families with 2 affected (at least 1 had bilateral breast cancer or breast cancer below age 50). The mean number of breast cancers per family was 3.4. Among the 617 probands with breast cancer, 104 women were diagnosed at age 40 years or below, 226 women were diagnosed between age 41 and 50 years, 200 women were diagnosed between ages 51 and 60 years, and 87 women were diagnosed above age 60. The mean age of breast cancer diagnosis among the 617 probands was 46 years (range of 28 to 76 years). Of the 617 patients, 81 also reported a family history of ovarian cancer and 16 reported a family history of male breast cancer. The 617 probands were selected from a registry of 3519 familial breast cancer cases housed at the Hereditary Cancer Center in Szczecin based on the number and age of onset of breast cancer cases among their relatives; all tested negative for a panel of 17 founder Polish mutations in *BRCA1*, *BRCA2*, *CHEK2*, *PALB2*, *NBN*, and *RECQL* [45]. All probands were ethnic Poles. 

### 2.2. Unselected Cases of Breast Cancer

We studied 12,484 prospectively ascertained cases of invasive breast cancer, diagnosed from 1996 to 2012, at 18 different hospitals in Poland (mean age of diagnosis 54.0 years, range 18–93). All women who were diagnosed with first primary invasive breast cancer at one of the participating centers were eligible. Patients with purely intraductal or intralobular cancer were excluded (DCIS or LCIS) but patients with DCIS with micro-invasion were included. Patients were unselected for family history. The patient participation rate among invited women was 76%. Information was recorded on the clinical characteristics of breast cancers through a review of medical records. Family history included the number of first- and second-degree relatives with any cancer and was available for 11,782 of 12,484 (93%) of cases. Of the 11,551 unselected cases, 1968 patients (17%) reported at least one first- or second-degree relative with breast cancer (familial breast cancer cases from unselected series). The definition of a familial case among the unselected cases was the presence of one or more breast cancers in a first- or second-degree relative (i.e., two or more breast cancers in first- or second-degree relatives in the family). Survival data were obtained (status: alive or dead, the date of death) from the Polish Ministry of the Interior and Administration in July 2014. The Ethics Committee of Pomeranian Medical University in Szczecin approved the study (IRB No. KB-0012/97/17). 

### 2.3. Controls

The purpose of the control group was to estimate with accuracy the *APOBEC3B* mutation frequency in the underlying Polish population. The control group included 3740 Polish cancer-free women between the ages of 18 and 94 years (mean age, 53.0 years). The controls were invited to participate, where they were interviewed and provided peripheral blood samples for DNA isolation between 2007 and 2013. In detail, these subjects were derived from four sources. The first series consisted of 987 women from the region of Szczecin (age range, 24 to 84 years) who were part of a population-based study of the 1.5 million residents of West Pomerania (North-West Poland) designed to identify familial aggregations of cancer, and these women were interviewed in 2007. The second group consisted of 1717 unselected women (age range 32–72) who participated in mammography screening at 8 different centers all over Poland between 2009 and 2011 (Kielce, Legnica, Olsztyn, Poznan, Szczecin, Swidnica, Torun, and Zielona Góra) and provided a blood sample for DNA analysis. The third control group included 1036 women (age range, 20–94 years) selected at random from the computerized patient lists of family practices located in the region of Opole (South Poland). These women were invited to participate by mail and participated in 2012 and 2013. 

### 2.4. Sequencing of the APOBEC3B Gene

We analyzed the entire coding sequence of *APOBEC3B* from the exome sequencing data of 617 women with hereditary breast cancer (step 1), as described previously [45]. The Agilent SureSelect human exome kit (V6) was used for capturing target regions. The regions were sequenced on Illumina NextSeq 500. The mean depth of coverage was approximately 100×, 97.4% of the CCDS exons were covered at 20× depth of coverage and higher which is used for variant calling. We looked for protein-truncating genetic variants, including frameshift insertions, deletions, stop codon mutations, and variants at the consensus splice sites, which are likely to be dysfunctional.

### 2.5. Genotyping

We genotyped 12,484 women with breast cancer and 3740 controls for the *APOBEC3B* mutation, which was detected by sequencing (step 2). DNA was isolated from 5 to 10 mL of peripheral blood. The c.783delG (p.Val262Phefs) mutation was genotyped using a TaqMan assay (Thermo Fisher Scientific, Waltham, MA, USA) in a LightCycler Real-Time PCR 480 System (Roche Life Science, Mannheim, Germany) using probes: APO-delG [HEX]CTTCTTGGACCTGGTTC[BHQ1] and APO-wt [6FAM] CTTCTTGGACCTGTTCC[BHQ1] and primers APO-delG-F AAGAATCTTCTCTGTGGCTTTTTAC and APO-delG-R CGGGTCCAACTGCAAAGAA. All mutations were confirmed by Sanger sequencing. Sequencing reactions were performed using a BigDye Terminator v3.1 Cycle Sequencing Kit (Thermo Fisher Scientific) according to the manufacturer’s protocol using primers GGCTAAGAATCTTCTCTGTG and CAGGAGATGAACCAAGTGAC. Sequencing products were analyzed on an ABI Prism 3100 Genetic Analyzer (Thermo Fisher Scientific).

### 2.6. Statistical Analysis

The prevalence of the *APOBEC3B* c.783delG (p.Val262Phefs) allele was estimated in 12,484 breast cancer cases and 3740 cancer-free women. Odds ratios were generated from two-by-two tables. Women with breast cancer, with and without an *APOBEC3B* mutation, were compared for age at diagnosis and clinical features of the breast cancers. Statistical significance was assessed using Fisher exact test or Chi-squared test where appropriate. Means were compared using the *t*-test. To estimate the survival of women with and without the mutation, we followed the breast cancer patients from the date of diagnosis until the date of death or July 2014. We compared the survival between mutation carriers and non-carriers by log-rank test. An age-adjusted hazard ratio was calculated using Cox regression analysis.

## 3. Results

We identified a truncating mutation in four of 617 women (0.61%) with hereditary breast cancer by sequencing the entire *APOBEC3B* gene. All four patients had the c.783delG (p.Val262Phefs) truncating mutation.

The c.783delG (p.Val262Phefs) variant was genotyped in 12,484 women with unselected breast cancer and 3740 cancer-free controls. It was found in 60 (0.48%) unselected cases and in 19 (0.51%) controls (OR = 0.95, 95% CI 0.56–1.59, *p* = 0.94). The mutation was detected in 8 of 1968 patients (0.41%) with familial breast cancer within the unselected cases (OR = 0.80, 95% CI 0.35–1.83, *p* = 0.74). The mutation frequency was identical (0.48%) in women diagnosed at the age of 50 years or below (28 of 5778) and in patients diagnosed above the age of 50 years (32 of 6706). The mutation frequency by age and family history of breast cancer is shown in Table 1.

We compared the clinical characteristics of the patients with breast cancer with and without the *APOBEC3B* mutation (Table 2). There were no statistically significant differences between mutation carriers and non-carriers in the age of diagnosis, tumor histology, tumor size, lymph node status, ER, PR, and HER2 receptor status. 

Survival data were available for 12,353 breast cancer cases. After a mean follow-up of 64 months, there were 10 deaths in 59 carriers of the *APOBEC3B* mutation (16.9%) and 2044 deaths in 12,294 non-carrier patients (16.6%) (HR = 0.99, 95% CI 0.53–1.84, *p* = 0.98; log-rank test). The 10-year survival was 78% for the carriers compared to 76% for non-carriers. The age-adjusted HR for mortality given the *APOBEC3B* mutation was 1.00 (95% CI 0.54–1.86; *p* = 1.00; Cox regression analysis).

We then analyzed the pedigrees of breast cancer patients with the *APOBEC3B* mutation to see whether or not there is an excess of different cancers in first- and second-degree relatives. No particular cancer was seen in excess in the relatives of the *APOBEC3B* mutation carriers, compared to those of non-carriers, including cancers of the breast, prostate, colon, kidney, lung, larynx, pancreas, stomach, cervix, endometrium, ovary, leukemia and lymphoma (Table 3).

## 4. Discussion

To date, over twenty genes have been associated with a genetic predisposition to breast cancer, but based on the mutation frequencies, the magnitude of cancer risk, and the influence on clinical management with mutation carriers, we consider that the most important breast cancer susceptibility gene to be *BRCA1*, *BRCA2*, *CHEK2*, and *PALB2*. The two major high-risk susceptibility genes *BRCA1* and *BRCA2* are routinely screened in familial cases for over two decades [46]. It is established that carriers of *BRCA1* and *BRCA2* mutations benefit from magnetic resonance imaging (MRI) of the breast, preventive mastectomy, prophylactic oophorectomy, and women with breast or ovarian cancer and a *BRCA1/2* mutation benefit from individualized chemotherapy using platins and PARP1 inhibitors [47,48,49,50]. *CHEK2* mutations confer about a 3- to 5-fold increased risk of breast cancer in a woman depending on her family history of breast cancer. *CHEK2* mutation-associated breast tumors are more likely to be estrogen receptor (ER)-positive than sporadic breast cancers, and patients with breast cancer who carry a *CHEK2* mutation respond well to tamoxifen [51,52,53,54]. Recently, we reported that oophorectomy improves the 15-year survival of breast cancer patients with a *CHEK2* mutation by about 15% and should be considered in the treatment of *CHEK2* mutation-associated breast cancers. The effect of oophorectomy was strongest in women with early-onset breast cancer (50 years or below) and those with adverse prognostic factors [52]. *PALB2* mutations confer about a 5-fold increase in the risk of breast cancer and about a 3-fold increase in the risk of ovarian cancer [55,56]. In our previous large study, two *PALB2* founder mutations (c.509_510delGA and c.172_175delTTGT) were associated with a 4.5-fold increased risk of breast cancer [56]. We have reported the prognosis of breast cancer associated with a *PALB2* mutation to be poor. For *PALB2* mutation carriers breast MRI is recommended and preventive mastectomy and oophorectomy should be discussed [56,57,58].

Poland is a genetically homogenous country, and this is expressed by the high frequency of founder mutations. Previously, we have identified pathogenic mutations of *BRCA1*, *BRCA2*, *CHEK2*, and *PALB2* in about half of 1018 Polish families with hereditary breast cancer, and we have observed that 18 founder mutations are responsible for about 80% of all the mutations detected in these genes [45]. Based on our results, in Poland, we use a panel of founder mutations in *BRCA1*, *BRCA2*, *CHEK2*, and *PALB2* as an initial screening tool for all patients with breast cancer and unaffected individuals with a positive family history of breast cancer. The advantages of testing for founder mutations include quick turnaround time and low cost. In patients who are found not to carry a founder mutation, we perform full sequencing using gene panels including *BRCA1*, *BRCA2*, *CHEK2*, and *PALB2* in breast cancer cases with familial clustering of this cancer and/or in patients with early onset disease (diagnosed at age 45 or below). In addition, we offer *TP53* sequencing for women with breast cancer diagnosed at the age of 30 or below, as it was reported that about 5% of these women carry a *TP53* mutation, and radiotherapy is contraindicated for *TP53* mutation positive cases [59]. Such a sequential testing approach could be also used in other homogeneous groups, i.e., Ashkenazi Jewish, Icelandic individuals, and French-Canadians, in which the range of mutations is limited [60]. However, it is not possible to apply this approach in genetically heterogeneous Western European countries or in North American populations where the background genetic variation is wide and common founder alleles are not present. In heterogeneous populations, genetic testing is usually based on NGS multigene genetic testing panels that include breast cancer susceptibility genes. 

To optimize genetic testing it is necessary to describe the full spectrum of breast cancer predisposing mutations in different populations, and to identify all breast cancer susceptibility genes. In the current study, we asked whether or not *APOBEC3B* mutations are present in Poland and whether they predispose to breast cancer. The *APOBEC3B* gene encodes an activation-induced cytidine deaminase (AICDA/AID), which is involved in the immune response against viruses, regulation of DNA methylation, and inducing DNA damage and apoptosis [31,32]. *APOBEC3B* has been described as a driver of breast cancer [33]. *APOBEC3B* expression is regulated by estrogen [61], a hormone critical in the pathogenesis of breast cancer. Somatic deletions of the *APOBEC3B* gene have been detected in breast cancer tumor tissue [36]. In addition, the germline *APOBEC3B* deletion of ~30 kb, which extends between the last exon of *APOBEC3A* and the eighth exon of *APOBEC3B* and removes the entire *APOBEC3B* protein-coding region, was associated with increased breast cancer risk in Asian populations [37]. 

In this study, we approached the question if *APOBEC3B* mutations predispose women to breast cancer in several ways, including the sequencing of the entire *APOBEC3B* gene in familial cases, and conducted a large case–control study and a pedigree analysis. We also investigated whether or not breast cancers in women with *APOBEC3B* mutations are associated with specific clinical characteristics and survival among patients. In none of these analyses was there evidence of cancer predisposition for carriers of an *APOBEC3B* truncating mutation.

Notably, we identified a single truncating mutation of the *APOBEC3B* gene by sequencing 617 hereditary cases (c.783delG, p.Val262Phefs). In a large case–control study, we showed that the c.783delG founder mutation was not associated with an increased risk of breast cancer in Polish women (OR = 0.95, *p* = 0.94). The mutation frequency was the same (0.48%) in 5778 women diagnosed at the age of 50 years or below and in 6706 patients diagnosed above the age of 50. These results contrast with those reported by Radmanesh et al., who published that the same c.783delG mutation of the *APOBEC3B* gene confers an increased risk of unselected breast cancer by 2.3 fold (95% CI 1.04–5.03, *p* = 0.04) and early onset breast cancer risk (below age of 50) by 3.2 fold (95% CI 1.37; 7.56, *p* = 0.007) in Belarus, Russia, Germany, and Iran [62]. There are several explanations for the discrepancy between our results and those of Radmanesh et al. [62]. It is likely that the difference is by chance. It is not likely that the difference was due to different mutations because the same *APOBEC3B* p.Val262Phefs mutation was analyzed in both studies. In our study, we identified a total of 83 mutation carriers, compared to 33 in the previous study. Based on our study there is insufficient evidence that the *APOBEC3B* p.Val262Phefs truncating mutation predisposes to breast cancer.

The c.783delG mutation results in a frameshift within exon 6 and creates premature termination of the protein 42 codons downstream (p.Val262PhefsTer42, NM_004900). It is located adjacent to the active site at Glu255 and eliminates the metal-binding sites of APOBAC3B, and therefore is predicted to lead to a loss of enzymatic function. In our study, the mutation was detected with an allele frequency of approximately 0.3% in Poland. The mutation has been reported by the Exome Aggregation Consortium (ExAC) at an overall MAF of 0.8%, with the highest prevalence in the African/African American population (5%), and much lower frequencies in European populations (0.3%), Ashkenazi Jews (0.3%) and the South Asian population (0.8%). Our study does not exclude a modest increase in breast cancer risk associated with the c.783delG allele and further studies in other ethnic groups are needed.

Our study is large and benefits from the genetic homogeneity of the Polish population which is populated by ethnic Slavs. The Polish population is well suited for association studies and false results due to admixture have not been reported. Our cases of breast cancer were not selected for family history. We have previously shown that the frequency of Polish founder alleles (i.e., in *BRCA1*, *CHEK2*, *NBN*, *RECQL* and *ATRIP*) is similar in different regions of Poland [26].

In summary, our results suggest that the *APOBEC3B* p.Val262Phefs founder mutation is not associated with an elevated risk of breast cancer in Poland. It was reported that a large deletion of the *APOBEC3B* gene of 30kb may confer a small increase in the risk of breast cancer risk in Asian populations, but it was not associated with breast cancer risk in non-Asian populations, including the Polish population [44]. It is unlikely that germline *APOBEC3B* mutations confer a clinically important risk of breast cancer (and probably other cancers). This data does not support the hypothesis that *APOBEC3B* is a cancer susceptibility gene.

## Figures and Tables

**Table 1:** Prevalence of the *APOBEC3B* c.783delG (p.Val262Phefs) mutation in 12,484 women with breast cancer, by age and family history of breast cancer in 3740 cancer-free women.

	Total (*n*)	*APOBEC3B* Mutation Positive	Prevalence (%)	OR (CI 95%)	*p*-Value
Patients with Breast Cancer					
All cases	12,484	60	0.48%	0.95 (0.56–1.59)	0.9
Age (years)					
≤40	1310	7	0.53%	1.06 (0.44–2.51)	0.9
41–50	4468	21	0.47%	0.93 (0.50–1.72)	0.9
51–60	3220	14	0.43%	0.86 (0.43–1.71)	0.8
61–70	2206	13	0.59%	1.16 (0.57–2.36)	0.8
≥71	1280	5	0.39%	0.77 (0.29 –2.06)	0.8
Number of Relatives with Breast Cancer *					
0	9583	46	0.48%	0.95 (0.55–1.61)	0.9
1	1510	5	0.33%	0.65 (0.24–1.75)	0.5
≥2	458	3	0.66%	1.29 (0.38–4.39)	0.9
Reference					
Cancer-free controls	3740	19	0.51%	-	-

Footnote: Odds ratios and *p* values calculated using cancer-free controls as a reference group. The distribution of genotypes was within the Hardy–Weinberg Equilibrium both among cases (*p* = 0.8) and controls (*p* = 0.9). * in first-degree or second-degree relatives.

**Table 2:** Clinical characteristics of breast cancers in carriers of the *APOBEC3B* c.783delG (p.Val262Phefs) mutation and in non-carriers.

	*APOBEC3B* Mutation Positive Cases *n* = 60	*APOBEC3B* Mutation Negative Cases *n* = 12,424	*p*-Value
Age at diagnosis (years)	53.5 (33–83)	54.0 (18–93)	0.7
Histological features			
Ductal, grade 3	7/52 (13.5%)	2023/10,093 (20%)	0.3
Ductal, grade 1–2	22/52 (42.3%)	4049/10,093 (40.1%)	0.9
Ductal, grade unknown	4/52 (7.7%)	669/10,093 (6.6%)	1.0
Medullary	0/52 (0%)	294/10,093 (2.9%)	0.4
Lobular	5/52 (9.6%)	1241/10,093 (12.3%)	0.7
Tubulolobular	2/52 (3.8%)	114/10,093 (1.1%)	0.2
DCIS with microinvasion	2/52 (3.8%)	304/10,093 (3%)	0.7
Other or undefined	10/52 (19.2%)	1399/10,093 (13.9%)	0.4
Receptor status			
Estrogen receptor-positive	36/43 (83.7%)	5963/8592 (69.4%)	0.1
Progesterone receptor-positive	35/44 (79.5%)	5882/8290 (70.9%)	0.3
HER2-positive	4/34 (11.8%)	1275/7243 (17.6%)	0.5
Size (cm)			
<1	7/46 (15.2%)	905/7929 (11.4%)	0.6
1–1.9	22/46 (47.8%)	3212/7929 (40.5%)	0.4
2–4.9	17/46 (37%)	3482/7929 (43.9%)	0.4
≥5	0/46 (0%)	330/7929 (4.1%)	0.3
Lymph node-positive	14/47 (29.8%)	3560/8179 (43.5%)	0.1
Bilateral	5/50 (10%)	448/9786 (4.6%)	0.1
Multifocal	5/49 (10.2%)	1068/8110 (13.2%)	0.7
Chemotherapy (yes)	24/50 (48%)	4923/9166 (53.7%)	0.5
Tamoxifen (yes)	29/37 (78.4%)	4116/6150 (66.9%)	0.2
Vital status (deceased)	10/59 (16.9%)	2044/12,294 (16.6%)	0.9

Footnote: data represent mean (range) or number/total (%), *p*-values compare mutation positive to mutation negative patients and were calculated using Fisher’s exact test; DCIS = ductal carcinoma in situ.

**Table 3:** Cancers reported in the families of the 54 unselected breast cancer cases with the *APOBEC3B* c.783delG (p.Val262Phefs) mutation compared to those reported by the 11,497 non-carrier cases.

Cancer Site	Number (%) of Cancers in Relatives of *APOBEC3B* Mutation Positive Patients (*n* = 54 Families)		Number (%) of Cancers in Relatives of *APOBEC3B* Mutation Negative Patients (*n* = 11,497 Families)		*p*-Value
	*N*	%	*N*	%	
Breast	9	16.7%	1904	16.6%	1.0
Colon	1	1.9%	891	7.8%	0.2
Kidney	2	3.7%	310	2.7%	1.0
Larynx	3	5.6%	454	3.9%	0.8
Lung	6	11.1%	1688	14.7%	0.6
Leukemia or Lymphoma	2	3.7%	462	4.0%	0.9
Pancreas	2	3.7%	322	2.8%	0.7
Prostate	4	7.4%	788	6.9%	0.9
Stomach	1	1.9%	968	8.4%	0.1
Cervix/Endometrium	1	1.9%	1179	10.3%	0.1
Ovary	2	3.7%	389	3.4%	0.9

Footnote: Cancer family history refers to tumors diagnosed in first- and second-degree relatives of breast cancer patients. Family history data were available for 54 of 60 *APOBEC3B* mutation carriers and 11,497 of 12,434 non-carriers.

## Data Availability

The main research data supporting the results of this study are included in Table 1, Table 2 and Table 3. As per the consent obtained from the Polish studied subjects, we are not able to share their individual sequence data with any third parties. Other data such as mutation frequencies generated for this study could be shared upon request.
